# Explicit criteria for prioritization of cataract surgery

**DOI:** 10.1186/1472-6963-6-24

**Published:** 2006-03-02

**Authors:** José Ma Quintana, Antonio Escobar, Amaia Bilbao

**Affiliations:** 1 Unidad de Investigación, Hospital de Galdakao, Barrio Labeaga s/n, 48960 Galdakao, Vizcaya, Spain; 2 Unidad de Investigación, Hospital de Basurto, Bilbao, Vizcaya, Spain; 3 Fundación Vasca de Innovación e Investigación Sanitarias (BIOEF), Sondika, Vizcaya, Spain

## Abstract

**Background:**

Consensus techniques have been used previously to create explicit criteria to prioritize cataract extraction; however, the appropriateness of the intervention was not included explicitly in previous studies. We developed a prioritization tool for cataract extraction according to the RAND method.

**Methods:**

Criteria were developed using a modified Delphi panel judgment process. A panel of 11 ophthalmologists was assembled. Ratings were analyzed regarding the level of agreement among panelists. We studied the effect of all variables on the final panel score using general linear and logistic regression models. Priority scoring systems were developed by means of optimal scaling and general linear models. The explicit criteria developed were summarized by means of regression tree analysis.

**Results:**

Eight variables were considered to create the indications. Of the 310 indications that the panel evaluated, 22.6% were considered high priority, 52.3% intermediate priority, and 25.2% low priority. Agreement was reached for 31.9% of the indications and disagreement for 0.3%. Logistic regression and general linear models showed that the preoperative visual acuity of the cataractous eye, visual function, and anticipated visual acuity postoperatively were the most influential variables. Alternative and simple scoring systems were obtained by optimal scaling and general linear models where the previous variables were also the most important. The decision tree also shows the importance of the previous variables and the appropriateness of the intervention.

**Conclusion:**

Our results showed acceptable validity as an evaluation and management tool for prioritizing cataract extraction. It also provides easy algorithms for use in clinical practice.

## Background

Cataract extraction is the most frequently performed surgical intervention in developed countries [1]. Because of the high prevalence of cataract development and the increasing number of patients requiring cataract extraction, managers must decide how to prioritize patients. Different priority criteria for cataract extraction have been created in the past in different countries, but none has included the appropriateness of the intervention [2-5].

The RAND-UCLA group in the United States [6] developed methodology to establish explicit criteria in the 1980s. This is a very popular methodology that has been used for many different diagnostic and therapeutic procedures since that time and lately for different applications [7-9].

The purpose of the current study was to develop priority criteria, using the RAND method, for patients undergoing cataract extraction and to consider the appropriateness of the indication.

## Methods

### Explicit criteria development

We based our development of priority criteria on a modification of the RAND appropriateness method. We first developed criteria to measure the appropriateness of the use of cataract surgery, according to the following steps.

First, an extensive literature search was performed to summarize existing knowledge concerning efficacy, effectiveness, risks, costs, and opinions about the use of phacoemulsification for cataract extraction.

Second, from this review, a comprehensive and detailed list of mutually exclusive and clinically specific scenarios (indications) was developed in which cataract surgery might be performed using phacoemulsification. Regarding cataract surgery, these indications included the following variables: presence of ocular comorbidities (simple cataract, cataract with diabetic retinopathy, or cataract with other ocular pathologies that may affect the visual prognosis), visual acuity in the cataractous eye and the contralateral eye, visual function, expected visual acuity after surgery, surgical technical complexity, and type of cataract. A total of 765 indications resulted from all possible combinations of the variables described previously and the respective categories. A description of all variables and their categories was reported previously [10].

Third, we compiled a national panel of expert ophthalmologists who were nationally recognized specialists in the field. Their names were provided by their respective medical societies and members of our research team.

The appropriateness ratings were confidential and took place in two rounds, using a modified Delphi process. Cataract surgery for a specific indication was considered appropriate if the panel's median score was between 7 and 9 without disagreement, inappropriate if the value was between 1 and 3 without disagreement, or uncertain if the median rating was between 4 and 6 or if the members of the panel disagreed. Disagreement was defined as occurring when at least one third of the panelists rated an indication from 1 to 3 and at least another third rated it from 7 to 9.

**Figure 1:**
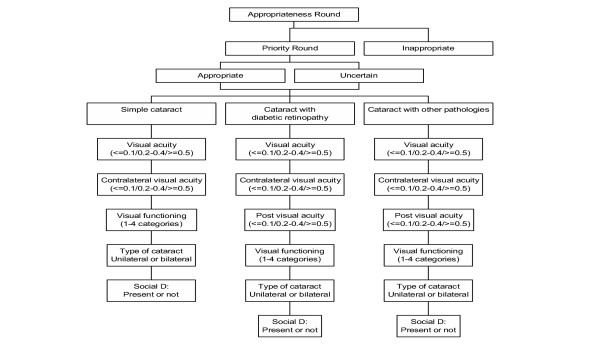
Variables in the priority algorithm. Social D: Social Dependence.

In a third round, we selected the scenarios judged in the second round as appropriate or uncertain. We selected the following previous variables for this priority round: appropriateness, presence of ocular comorbidities, visual acuity in the cataractous eye, visual function, visual acuity in the contralateral eye, expected visual acuity after the intervention, the type of cataract (laterality), and the new variable social dependence. Selection of the variables was based on the review of the bibliography and the research team best judgment. Figure 1 shows the variables included in this priority round and Appendix 1 includes a definition of all variables and their categories.

We requested that the same panelists score the 310 scenarios. Ratings also were scored on a 9-point scale, with 9 indicating the highest priority and 1 the lowest compared to other scenarios. Priority in the context of cataract extraction was defined as the benefit that the patient may obtain from undergoing surgery. The higher the benefit was for the patient, for a similar risk of complications, the higher the priority of the intervention was. Benefit was defined in terms of quality-of-life improvement. Three categories, from higher to lower priority, were established. The priority of cataract surgery was considered high for a specific indication if the panel's median rating was between 7 and 9 without disagreement, low if the value was between 1 and 3 without disagreement, or intermediate if the median rating was between 4 and 6 or if the panel members disagreed. Disagreement was defined as occurring when at least one third of the panelists rated an indication from 1 to 3 and at least another third rated it from 7 to 9. This method did not attempt to force panelists to reach agreement on the priority.

To determine the use of all theoretical indications created in clinical practice, data related to the algorithm variables were gathered for 936 patients on a waiting list to undergo cataract extraction exclusively by phacoemulsification from the ophthalmologic services of six hospitals of our area. The number of theoretical indications used in clinical practice was calculated for each of the three main diagnostic groups; simple cataract, cataract with diabetic retinopathy, or cataract with other ocular pathologies that may affect the visual prognosis.

### Statistical analysis

We estimated the mean priority rating of all indications for each panellist and the median and the deviation from the panel mean. We created a continuous priority score, which is the sum of the ratings of the 11 panelists, standardised to a 0 to 100-point scale.

Determinants of priority scores and their contribution to the model explanation were assessed with the general linear model [11], with the priority score the dependent variable and the variables in the algorithm the covariates. Ordinal logistic regression also was used, which was the classification of the panelists' scores in the categories of high, intermediate, or low priority of the dependent variable (12). In each model, we studied the degree of variability explained by each variable by means of the R-square and -2 log L statistics, respectively. Because the appropriateness variable is a combination of some of the other variables, we forced it to the enter the last in both models.

We used two methods to determine priority coefficients for the categories of each variable that should permit rapid estimation of a priority score in practice. In the first model, the optimal scaling method [13,14] considered the priority weighted score as the dependent variable and the variables in the algorithm as the independent variables. Optimal scaling was used to quantify the categorical data by assigning numerical values to the categories in such a way that the fit is optimal. Standard linear regression analysis then was conducted using the numerical explanatory variables obtained with the optimal scaling method. The weights for each variable were calculated based on the t-values from linear regression, with weights distributed across variables as the corresponding proportion of the total model t-value. To determine the weights for the categories within each variable, the numerical values for the categories obtained with the optimal scaling method were rescaled linearly to between 0 and 1; these values then were multiplied by the weight for the variable to which the levels belong.

In the second model, we performed the same estimation using a general linear model in which we created dummy variables from the categories of the independent variables, with the priority score the dependent variable. Weights for categories within each variable were based on beta values from the general linear model, with weights transformed to a 0 to 100 point scale distributed across variables as the corresponding proportion.

Therefore, in both cases weights are apportioned among variables so that the scores range from 0 to 100. The scores obtained with both methods for all scenarios were compared with the original panel priority scores using Pearson and Spearman correlation coefficients.

Algorithms in decision-tree form were compiled by means of classification and regression trees (CART) analysis [15]. CART was used to build a regression tree with a dependent variable as the priority score.

All statistical analyses were performed using the SAS for Windows, version 8, except for CART analysis in which we used S-Plus 2000 software (MathSoft Inc., 1999), and the optimal scaling analysis in which we used SPSS v.12 statistical software.

## Results

**Figure 2:**
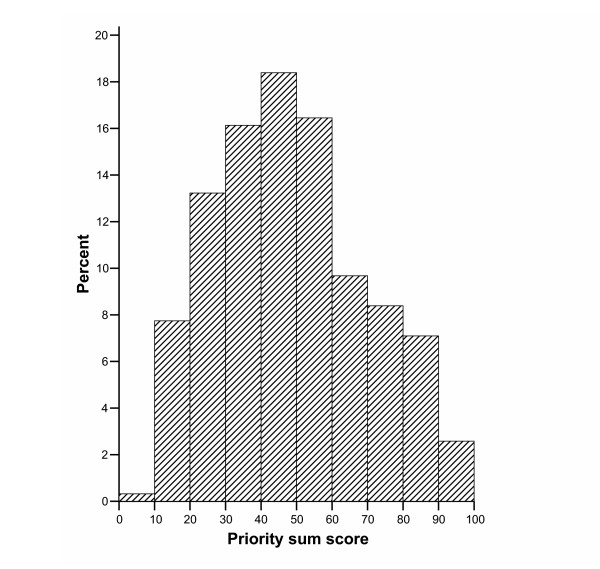
Distribution of panelists' scores.

A panel of 11 experts scored 310 scenarios. Figure 2 shows the panel scores for the priority score as a continuous variable. The histogram resembles a normal plot and fulfills normality (p = 0.12 by Kolmogorov-Smirnov test). The panelists' scores tended to be similar (Table 1), except for panelists 5, and 6, who had the extreme high and low mean and median scores.

Table 2 shows the classification of the panelists' scores in three categories from high to low priority. Of the 310 indications they evaluated, 22.6% had a high priority, 52.3% intermediate priority, and 25.2% low priority. Agreement was reached for 31.9% of the indications and disagreement for 0.3% (Table 2).

We evaluated the contribution of each variable included in the algorithm by means of general linear and logistic regression models (Table 3). Globally, the general linear model indicated that the most influential variables were the visual acuity in the cataractous eye, the anticipated visual acuity postoperatively, and the visual function. Social dependence and the type of cataract had the lowest effect, although, in all cases, the effect reached statistical significance.

The logistic regression model indicated that the same three variables were the most important but in a different order, i.e., the visual function, the visual acuity in the cataractous eye, and the anticipated postoperative visual acuity. The other variables were not statistically significant in this model. Appropriateness of the intervention, despite being forced to enter in the model after the rest of the variables to avoid collinearity, remained highly significant and fourth in the order of importance in both models.

We present two alternative methods of obtaining a simpler way to obtain the priority score: optimal scaling and general linear models (Table 4). The anticipated postoperative visual acuity and the visual acuity in the cataractous eye correspond to the highest weights (21 points each), followed by visual function, appropriateness, and the visual acuity in the contralateral eye. The presence of ocular comorbidities, social dependence, and laterality had less than 10 points. The weights obtained using the general linear model were similar and in the same order. In addition, these methods give different distances among categories within a variable.

We studied the correlation of the scores obtained between the two scoring methods with the priority scores of the original panel. The Pearson correlation coefficient was 0.95 for the optimal scaling model and 0.97 for the general linear model. Using the Spearman correlation coefficient, the results were 0.94 and 0.97, respectively.

**Figure 3:**
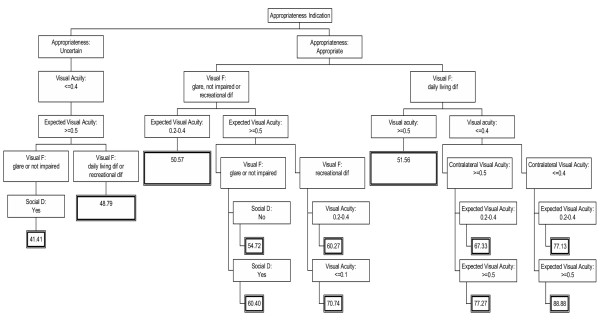
Priority decision tree. The decision tree was developed using regression tree analysis (CART analysis) with the dependent variable the priority sum score. The number in the final box indicates the priority score for that scenario. Priority scored can ranges from 0 to 100; the higher the number, the higher the priority. Social D: Social Dependence, Visual F: Visual Function.

Finally, the criteria developed by the panel of experts were summarized by CART analysis on a decision regression tree (Figure 3), which shows that the highest priority was given to the scenarios considered first as appropriate. This is followed by visual function (difficulties with daily activities), visual acuity in the cataractous eye (= 0.4), visual acuity in the contralateral eye, and the anticipated postoperative visual acuity.

We collected information on 936 patients to identify which of the theoretical indications were used in clinical practice. Globally, 102 of 310 theoretical (33%) indications were assigned to 936 patients. By diagnostic group, for simple cataract, 53 of 86 theoretical (62%) indications were assigned to 761 patients with that diagnosis. Of those with diabetic retinopathy, 11 of 118 theoretical (9%) indications were assigned to 19 patients. Of those with other ocular pathologies, 38 of 106 theoretical (35.8%) indications were assigned to 156 patients with that diagnosis.

## Discussion

Cataract extraction has been chosen by different researchers to develop priority scoring systems of [2,5,16]. The drawback of all systems is that they do not take into account the appropriateness of the intervention as a previous consideration. Our study incorporates this variable on the priority algorithm, and we show that it plays an important role in patient prioritization.

To develop our system of prioritization, we used the RAND methodology. Although the RAND methodology was developed primarily to create appropriateness explicit criteria, it also can be used for other purposes (9;17;18). To the best of our knowledge, this is the first time the RAND methodology has been used to develop priority criteria linked with previous appropriateness criteria.

We first determined the appropriateness of the indication criteria in two panel rounds. We then selected those indications ultimately classified as appropriate or uncertain. The fact that the appropriate indications were entered in the prioritization algorithm seems logical; however, why should uncertain surgical indications be included? First, because as in some studies in which appropriateness had been related to outcomes, the outcomes of the appropriate and uncertain cases seemed similar [19,20]. Uncertainty does not indicate that the intervention should not be performed, but rather that either that the panel did not reach agreement or that the panel's opinion is that there is uncertainty about whether the intervention is worthwhile because of lack of evidence or panel experience or ambiguous results. Second, we considered that social dependence, a new variable in our algorithm, might result in an uncertain scenario being considered as appropriate. As other authors who had worked with this methodology pointed out, other circumstances not included in the appropriateness criteria may play an important role in clinician decision making for indicating an intervention. More RAND appropriateness criteria had in common that they include clinically relevant variables but not socially relevant variables or those important to the patient [21]. We considered that social dependence is important in many clinical situations but mainly to prioritize patients. Focus groups performed by our group and other groups studying cataract have shown that patients believe that social dependence is much more relevant than other clinical variables [22]. But also because social dependence has been included in previous priority scoring systems [2,5].

We initially spent considerable effort defining what we consider priority. Priority differs depending on the health problem and its probability of leading to death or important permanent handicaps if untreated. That introduces the concept of urgency, i.e., if not treated promptly death will result [23], as with myocardial infarction or other cardiovascular problems, in which the risk of death or incapacity is often present. However, cataract itself never implies the risk of death for the patient. In this case, we then defined priority in terms of the benefit of the intervention to the patient. The higher the benefit for the patient, with similar low risks, the higher the priority of the intervention. We understand the benefit as the highest gain in the quality of life or living or working independently for the patient, which would be correlated with relevant improvements postoperatively in their ability to function. With cataract, this improvement should not only correlate with improved visual acuity (either measured by Snellen or lines of improvement or changes in the log MAR visual acuity), as reported by the ophthalmologist, but also with a relevant improvement in visual function, as reported by the patient. If a patient with a preoperative visual acuity of 0.3 and another with 0.1 or less are expected to have improvements in visual acuity to 1.0 and 0.7, respectively, with the rest of their circumstances equal, who should undergo surgery first? The absolute gain in the first case (0.7) is superior to the second (0.6); however, considering functionality, while the visual acuity of the first patient probably imposed limitations, he or she likely could perform some daily activities but was fully functional after surgery. The second patient, who would be legally blind in our country, has a visual function much poorer than the first patient; therefore, a visual acuity improvement to 0.7 may allow him or her to manage daily activities or live independently. The improvement in the second patient has greater relevance considering the quality of life.

As we pointed out previously, most priority criteria developed thus far did not consider the appropriateness of the intervention. Some may assume that all patients on the waiting list are appropriate candidates, which it is not necessarily true. Our results from both the general linear model and the CART analysis indicated that appropriateness was an important variable for the panelists when scoring the priority of each scenario. As reported previously, the rate of inappropriate interventions ranges from 1.3% [24] to 7.7% [25] depending on the study. Although compared to other appropriateness RAND studies those rates are relatively low [24-26], an inappropriate intervention should not be considered for prioritization. However, in the previous studies, uncertain indications ranged from 7% to 36.8%. Because they occur much more frequently, they should be considered carefully.

To the best of our knowledge, one recent study attempted to match appropriateness and priority [3]. However, although the development of appropriateness criteria followed RAND methodology similar to ours, the development of the priority criteria differed and resembled previous studies. Other studies in Canada [2,16] and New Zealand [27] developed priority criteria by different methods, some of which have been questioned (4). The Canadian group used similar statistical methodology (optimal scaling) to generate a simpler scoring system [2,16]. In any case, all of them share similar variables in their priority algorithm as ours.

We presented the results of the panel scoring as a categorical variable or continuous variable. To construct the categorical variable, with three categories of priority (from high to low), we used the same algorithm to develop the three RAND categories of appropriateness (appropriate, uncertain, inappropriate). An advantage is that this system may match current priority setting norms in some places such as ours. A disadvantage is that it can seem artificial, or with no conceptual base, to have those categories. On the other hand, the continuous variable provides a priority order for all scenarios, but it can seem less practical. Daily use will determine which one is more useful.

On the other hand, to have an easily usable algorithm, we presented our results simply. We presented two alternative priority scoring systems, one developed by optimal scaling and the other by a classical general linear model. The importance of the variables and the weights assigned to the different categories of variables were similar. In addition, both had very high Pearson and Spearman correlations with the original panel scores. An alternative way to use the real panel scores can be provided by simple software that allows the final score to be obtained by clicking on the categories of each variable. We are working on this alternative method.

Finally, we presented a decision tree that provides a global idea of which clinical situations should be treated first and indicates the importance of assessing the appropriateness of the intervention for prioritization.

In conclusion, this is the first study to combine the appropriateness of an indication with priority criteria following the same methodology. Our results show that appropriateness plays an important role in priority decision making process. The variables included also were identified by other authors, which adds validity to our criteria. We also provide alternative scoring algorithms that allow ease of use of our criteria in clinical practice. Use of them will let us know which one is more useful. Nevertheless, more sophisticated future proposals should include the algorithm in flow models [28] that allows management of patients placed on a waiting list.

## Appendix 1

Variables included in the prioritization algorithm.

1. Presence of ocular comorbidities.

a. No presence or simple cataract. Patients who do not present with any other ocular pathology in the operated eye that may affect the visual prognosis. This includes glaucoma controlled by medication or surgery without deteriorating central vision, myopia without retinopathy, and vascular occlusion not affecting central vision.

b. Cataract with diabetic retinopathy, according to the Early Treatment Diabetic Retinopathy Study classification. This includes nonproliferative diabetic retinopathy, proliferative diabetic retinopathy without high-risk characteristics, cases in which only phacoemulsification will be performed, and the absence of macular edema.

c. Cataract with other ocular pathologies that may affect visual prognosis. This includes retinopathies: retinal detachments that have been treated surgically, retinitis from any cause; maculopathies: macular degeneration due to toxicity (alcohol), medications, hereditary factors, extreme myopia, age-related macular degeneration; vasculopathies: branch retinal vein occlusion, venous thrombosis; neuropathies: optical neuritis and glaucomatous neuropathy; amblyopia; corneal opacity or corneal dystrophies (except Fuchs' dystrophy): leukoma without keratoplasty indication.

2. Preoperative visual acuity in the cataractous eye. Best-corrected visual acuity measured by Snellen optotypes. Three visual acuity categories :≥ 0.5; 0.2–0.4; = 0.1

3. Visual acuity preoperatively in the contralateral eye. Similar to the previous category.

4. Visual function. Effect of cataract on the quality of life of the patient. Four categories:

a. Unimpaired. The patient reports no impairment in visual function.

b. Glare (visual discomfort). Visual perception diminished depending on light intensity (outside or inside).

c. Recreational difficulties. Visual difficulties with activities that do not affect patient autonomy, e.g., watching TV or reading.

d. Difficulties with activities of daily living. Activities that affect patient autonomy, including glare when driving if this affects the patient's work.

5. Expected visual acuity postoperatively. A subjective judgment of the ophthalmologist before the intervention. This is the visual acuity that the patient is expected to reach after surgical intervention based on the preoperative ophthalmologic examination. Three visual acuity categories: ≥ 0.5; 0.2–0.4; = 0.1.

6. Social dependence. Situation in which the patient is dependent on a relative, friend, or other care giver for help with the activities of daily living.

7. Laterality of cataract. Unilateral or bilateral cataract.

8. Appropriateness. Appropriate or uncertain indication based on previously developed explicit criteria.

## Competing interests

The author(s) declare that they have no competing interests'

## Authors' contributions

JMQ conceived of the study, coordinated and participated in the design of the study, and drafted the manuscript. AE participated in the design and helped to draft the manuscript. AB performed the statistical analyses and drafted the manuscript. The IRYSS-Appropriateness Cataract Group participated in the design of the study and helped to draft the manuscript. All authors read and approved the final manuscript. The IRYSS-Appropriateness Cataract group included the following co-investigators: Dr. Jesús Martínez-Tapias, Dr. Eduardo Aguayo (Hospital Universitario Virgen de las Nieves, Granada); Dr. Emilio Perea-Milla (Hospital Costa del Sol, Málaga); Dr. Juan Ramón Lacalle (Universidad de Sevilla); Dra. Marisa Baré, Dra. Gemma Navarro (Corporació Sanitaria Parc Taulí, Sabadell); Dra. Elena Andradas, Dr. Juan Antonio Blasco (Agencia Laín Entralgo, Madrid); Inmaculada Arostegui (Departamento de Matemática Aplicada, UPV); Berta Ibáñez (Fundación Vasca de Innovación e Investigación Sanitarias); Dr. Txomin Alberdi (Servicio de Oftalmología, Hospital de Galdakao, Bizkaia); Dra. Sandra de Fernando (Servicio de Oftalmología, Hospital de Cruces, Bizkaia); Dra. Fabiola Eder (Servicio de Oftalmología, Hospital de Donostia, Gipuzkoa); Dr. Jose Ma Beguiristain, Dra. Belén Elizalde (Dirección Territorial de Gipuzkoa); Dra. Idoia Garai (Dirección Territorial de Bizkaia); Dr. Joseba Pérez de Arriba (Dirección Territorial de Alava); Dr. Jose Ignacio Pijoan (Unidad de Investigación del Hospital de Cruces, Bizkaia); Dr. Felipe Aizpuru (Unidad de Investigación del Hospital de Txagorritxu, Alava); Nerea González, Iratxe Lafuente, Urko Aguirre (Unidad de Investigación del Hospital de Galdakao, Bizkaia).

## Pre-publication history

The pre-publication history for this paper can be accessed here:



## Figures and Tables

**Table 1:** Panelists' scores

	*Score*			*Mean deviation*
		
*Panelist*	*Mean*	*SD*	*Median*	
1	5.0	1.8	5.0	0.09
2	5.4	2.2	5.5	0.47
3	4.3	2.0	4.0	-0.63
4	5.6	1.5	6.0	0.70
5	6.7	1.9	7.0	1.77
6	2.9	2.1	2.0	-1.99
7	4.9	2.3	5.0	-0.04
8	5.5	2.5	6.0	0.56
9	4.6	2.8	5.0	-0.27
10	4.6	2.0	5.0	-0.35
11	4.6	2.8	4.0	-0.32

SD, standard deviation.

**Table 2:** Agreement and priority judgment of the panel of experts

	*Priority*			
	
	*High*	*Intermediate*	*Low*	*Total*
Agreement	42 (60.0)	17 (10.5)	40 (51.3)	99 (31.9)
Undetermined	28 (40.0)	144 (88.9)	38 (48.7)	210 (67.7)
Disagreement	0 (0.0)	1 (0.6)	0 (0.0)	1 (0.3)
Total	70 (22.6)	162 (52.3)	78 (25.2)	310 (100.0)

**Table 3:** Contribution of the variables to the priority score

	*General linear model*				*Logistic regression*		
			
*Variable*	*R*^2^	*Difference*	*P value*	*Variable*	*-2 log L*	*Difference*	*P value*
					924.040		
Visual acuity in cataractous eye	0.136		<0.001	Visual function	824.363	99.677	<0.001
Expected visual acuity	0.390	0.254	<0.001	Visual acuity in cataractous eye	806.617	17.746	<0.001
Visual function	0.777	0.387	<0.001	Expected visual acuity	770.361	36.256	0.011
Contralateral visual acuity	0.824	0.047	<0.001	Contralateral visual acuity	766.778	3.583	0.250
Ocular comorbidities	0.872	0.048	<0.001	Ocular comorbidities	764.305	2.473	0.596
Social dependence	0.881	0.009	<0.001	Social dependence	762.447	1.858	0.160
Laterality	0.882	0.001	0.001	Laterality	762.418	0.029	0.843
Appropriateness	0.947	0.065	<0.001	Appropriateness	756.008	6.410	0.007

R^2^, R square. Difference, difference in R square after the introduction of a new variable.

**Table 4:** Prioritization scoring systems

		*Weights for each category*	
		
*Variable*	*Category*	*Optimal scaling*	*General linear model*
Visual acuity	= 0.1	**21**	**25**
	0.2–0.4	15	17
	≥ 0.5	0	0
Expected visual acuity	= 0.1	0	0
	0.2–0.4	13	18
	≥ 0.5	**21**	**29**
Visual function	Unimpaired	0	0
	Glare	4	5
	Recreational difficulties	10	11
	Daily living difficulties	**20**	**20**
Contralateral visual acuity	=.1	**10**	**7**
	0.2–0.4	6	5
	≥ 0.5	0	0
Ocular comorbidities	Simple cataract	**8**	**6**
	Diabetic retinopathy	4	3
	Other pathologies	0	0
Social dependence	Yes	**5**	**3**
	No	0	0
Laterality	Unilateral	0	0
	Bilateral	**2**	**1**
Appropriateness	Appropriate	**13**	**9**
	Uncertain	0	0

Scoring estimation: the sum of the score of the corresponding category of each of the eight variables. Final score can range from 0 to 100, the higher the score the higher the priority.
